# Healthcare Providers’ Awareness and Perceptions of Competency Requirements in Central Venous Catheter Insertion

**DOI:** 10.15694/mep.2018.0000012.1

**Published:** 2018-01-15

**Authors:** Elaine R. Cohen, Jeffrey H. Barsuk, Joelle R. Hertz, Diane B. Wayne, Yasuharu Okuda, Debi Mitra, William C. McGaghie, Kenzie A. Cameron

**Affiliations:** 1 Northwestern University Feinberg School of Medicine; 2 Eastern Washington University; 3 The Simulation Learning

**Keywords:** competency, central venous catheter, mastery learning, simulation, qualitative research

## Abstract

This article was migrated. The article was marked as recommended.

Background

Studies show that medical residents do not feel comfortable and lack the skills and confidence to perform common bedside procedures. Regulatory bodies often require a set number of procedures to determine resident competence, yet medical providers’ knowledge of competency guidelines are less well known. This study aimed to qualitatively assess existing practices relevant to documentation of competency in central venous catheter (CVC) insertion and explore healthcare providers’ awareness and perceptions of those practices at their institutions.

Methods

The authors performed a qualitative study at Veterans Affairs Medical Centers (VAMCs) from February to December 2014 as part of a larger project related to the dissemination of a simulation-based CVC insertion curriculum. Two authors conducted interviews with hospital staff (including attending physicians, nurses, and residents) at selected VAMCs. Recordings of interviews were transcribed, coded, and analyzed using a grounded theory approach and constant comparative techniques.

Results

Twenty-six participants were interviewed at six VAMCs. Participants reported varying perspectives regarding their institutions’ policies about CVC insertion. Four major themes emerged: (1) knowledge of institutional policy; (2) competency by numbers; (3) documentation of competency; and (4) perceptions of competency measures. Participants reported concern about the reliability of these policies and measures of competence.

Conclusions

This study demonstrates that healthcare providers’ knowledge and perceptions about institutional requirements for procedural competency vary widely. Our findings suggest the need for establishment of consistent competency policies based on evidence-based practices, and highlight the need for increased communication regarding individual institutional policies. Integration of rigorous simulation-based education, implemented consistently across institutions, can provide a reliable mechanism to train and assess procedural competence and ensure patient safety.

## Background

Our current medical education system produces physicians with variable abilities to perform invasive procedures ( Barsuk, Ahya, Cohen, McGaghie, & Wayne, 2009; Barsuk et al., 2016; Bell, 2009; Birkmeyer et al., 2013; Wayne, Barsuk, O’Leary, Fudala, & McGaghie, 2008). Regulatory bodies are responsible for keeping patients safe and licensing clinicians in their role to have the knowledge and skills to perform competently. The Accreditation Council for Graduate Medical Education (ACGME) recently implemented the Milestones accreditation framework, requiring assessment of trainee performance through competency-based outcomes (“Accreditation Council for Graduate Medical Education Milestones”, 2016). Additionally, the American Board of Internal Medicine (ABIM) requires Internal Medicine residency program directors to verify trainee knowledge and understanding to perform specific procedures such as paracentesis, lumbar puncture, nasogastric intubation, thoracentesis and central venous catheter (CVC) insertion (“American Board of Internal Medicine Policies and Procedures for Certification”). However, despite these regulatory body requirements, studies have indicated that many medical residents and fellows still do not feel comfortable ( Berns, 2010; Hicks et al., 2000; Huang et al., 2006) and lack the skills ( Barsuk, Ahya, et al., 2009; Barsuk et al., 2012; Bell, 2009; Wayne et al., 2008) to perform common bedside procedures after completing graduate medical education.

As an example, CVC insertion is an invasive procedure where residents, fellows and attending physicians have consistently demonstrated uneven skills ( Barsuk, Ahya, et al., 2009; Barsuk et al., 2016; Barsuk, McGaghie, Cohen, Balachandran, & Wayne, 2009; Barsuk, McGaghie, Cohen, O’Leary, & Wayne, 2009; McQuillan et al., 2015). As CVC insertion is associated with life threatening complications including pneumothorax, arterial puncture, and central line-associated bloodstream infection, this skill variability has the potential to cause significant morbidity and mortality ( McGee & Gould, 2003). Yet, medical regulatory bodies continue to have varying guidelines for determination of competency. For instance, the ABIM specifies that “to assure adequate knowledge and understanding of the common procedures in internal medicine, each resident should be an active participant for each procedure [including CVC insertion] five or more times,” a number recommended based on expert consensus opinion (“American Board of Internal Medicine Policies and Procedures for Certification”, 2017). In the United Kingdom, the Joint Royal Colleges of Physicians Training Board (JRCPTB) expects trainees to be competent in CVC insertion for general internal medicine, but no specific required number of procedures is identified (“Specialty Training Curriculum for General Internal Medicine”, 2012). When considering requirements at the institutional level, many hospitals require a specific number of CVC insertions before granting privileges to attending physicians to perform the procedure on patients, yet these numbers appear arbitrary and inconsistent (“How many procedures makes competency?”, 2014). Hence, achievement of competency remains vague. Eventhough some organizations and institutions specify a required number of procedures to demonstrate competency, data supporting an optimal number of required procedures to demonstrate competency are lacking ( Barsuk, Ahya, et al., 2009; Barsuk et al., 2012; Barsuk, McGaghie, Cohen, Balachandran, et al., 2009; Barsuk, McGaghie, Cohen, O’Leary, et al., 2009; Hicks et al., 2000; Wayne et al., 2008).

In addition to the lack of consistent guidelines used to determine competence, medical providers’ knowledge and understanding of their institution’s definitions of competency are unclear (“How many procedures makes competency?” 2014). To begin to better understand the perceptions of healthcare providers engaged in medical education, this study aimed to qualitatively assess existing practices relevant to documentation of competency in central venous catheter (CVC) insertion. We also explored healthcare providers’ awareness and opinions of those practices at their institutions.

## Methods

### Setting and participants

We performed a qualitative study at Veterans Affairs Medical Centers (VAMCs) from February to December 2014 that was part of a larger project related to the dissemination and implementation of a CVC insertion simulation-based education course ( Barsuk et al., 2016). SimLEARN, the national simulation training and education program for the Veterans Health Administration (VHA), initially identified 60 VAMCs (from a final total of 123) to participate in a two-day train-the-trainer course. At least one instructor travelled to each site to train VAMC personnel how to deliver a CVC simulation-based mastery learning (SBML) curriculum at their institution. Simulation combined with mastery learning and deliberate practice ensures trainees are rigorously assessed before performing procedures on patients ( McGaghie, Issenberg, Barsuk, & Wayne, 2014). Personnel participating in the course (“trainers”) learned how to teach the SBML curriculum on Day 1 and were observed training and assessing “sample learners” on the second day. The curriculum has been described in further detail elsewhere ( Barsuk, Cohen, Feinglass, McGaghie, & Wayne, 2009; Barsuk et al., 2014; Barsuk, McGaghie, Cohen, Balachandran, et al., 2009; Barsuk, McGaghie, Cohen, O’Leary, et al., 2009).

A total of 236 healthcare providers from the initial 60 identified VAMCs participated in the course. To better understand providers’ existing perceptions and previous experiences regarding CVC insertion, we conducted semi-structured interviews with course participants (attending trainers and resident physician sample learners) immediately before participation in the course. We also interviewed Intensive Care Unit (ICU) nurses (who did not participate in the training) to broaden our understanding of institutional practices related to CVC insertion. We used stratified purposeful sampling ( Patton, 2001) to identify a sample of eight participating VAMCs from the first 60 site visits at which to conduct the interviews, selecting the sample to be both geographically distinct and to consist of VAMCs of varying sizes. All participants provided written informed consent before their participation in the interviews, which included consent to digitally record the interview for transcription. The Northwestern University and VAMC Institutional Review Boards approved this study (Northwestern IRB approval #STU00089190 and VA approval protocol #15-00034).

### Data collection

A multidisciplinary research team, including an academic hospitalist, proceduralist and simulation expert (JHB), a communication scholar and qualitative expert (KAC), a medical education researcher (ERC) and a general internist and medical education expert (DBW), developed a semi-structured interview guide. As part of understanding existing institutional procedural processes for CVC insertion, the guide included items designed to assess and explore participants’ awareness and understanding of these relevant competency guidelines (see Table 1). Probes were developed for use during the interviews to clarify responses. We pilot tested the interview guide with three individuals who participated in our first VAMC site visit, but were not included as part of the study sampling frame. The guide was revised based on feedback received. Two authors (KAC, JHB) performed the interviews at selected VAMCs until thematic saturation was reached; interviews were audio recorded.

**Table 1.**
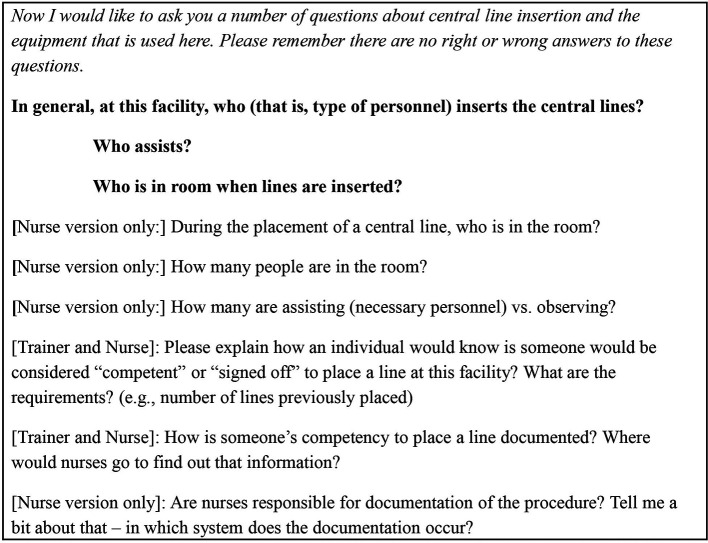
Interview Guide Questions Regarding Competency in CVC Insertion Practices *

^*^ Interview questions and probes differed slightly, based on the type of interviewee (course trainer, trainee, and ICU nurse). Questions that were asked of all participants are in **bold**. Variations based on type of interviewee are noted.

### Data analysis

Digital recordings of the interviews were transcribed verbatim by an external company and reviewed and coded by three authors (KAC, ERC, JRH) for accuracy. In the one case where an interviewee declined audio recording, detailed field notes were taken, with identification of direct quotations. Personal identifiers were removed and each transcript was coded and analyzed using a grounded theory approach and the constant comparative method ( Glaser, 1965; Kolb, 2012; Strauss & Corbin, 1998). The three coders reviewed all transcripts from one VAMC site to build a preliminary codebook. They independently assessed participant responses before convening to compare and compile findings. Coding of transcripts revealed a preliminary list of initial open codes; these codes were then grouped into higher order themes. The coders met multiple times to discuss and refine the identified themes and codes and to triangulate their perspectives ( Strauss & Corbin, 1998). Group meetings continued until consensus on the overarching themes and related codes was achieved. Using multiple coders is common to control for any subjective bias each coder brings to the analytic process ( Lincoln, 1985). After coders reached consensus on the codebook from the one VA site, each transcript from all sites was coded by two of the three coders. The coders for each transcript compared and compiled their individual findings, discussing any identified variations ( Glaser, 1965). In cases where the two coders were unable to reach initial consensus, the third coder was consulted. There were no cases where the coders were unable to reach final consensus. While multiple themes arose inductively from the coding process, this manuscript focuses on participants’ knowledge and awareness of existing practices regarding competency and documentation of competency in CVC insertion, as well as participants’ perceptions of identified competency requirements.

## Results

### Sample characteristics

Saturation was achieved after interviewing 26 participants across six sites ( Table 2). All VAMCs had an academic affiliation. As the larger project required sites to identify and provide “trainers” and “sample learners” to participate in the CVC SBML course, we had a variety of types of interview participants including 10 attending physicians, 6 resident physicians and 10 nurses. Individuals selected by their institutions to be trainers ranged from rising chief residents to physician champions for simulation. Nurses were primarily nurse managers of the medical or coronary intensive care units. Interviewed learners ranged from residents to nurse practitioners to staff physicians.

**Table 2.**
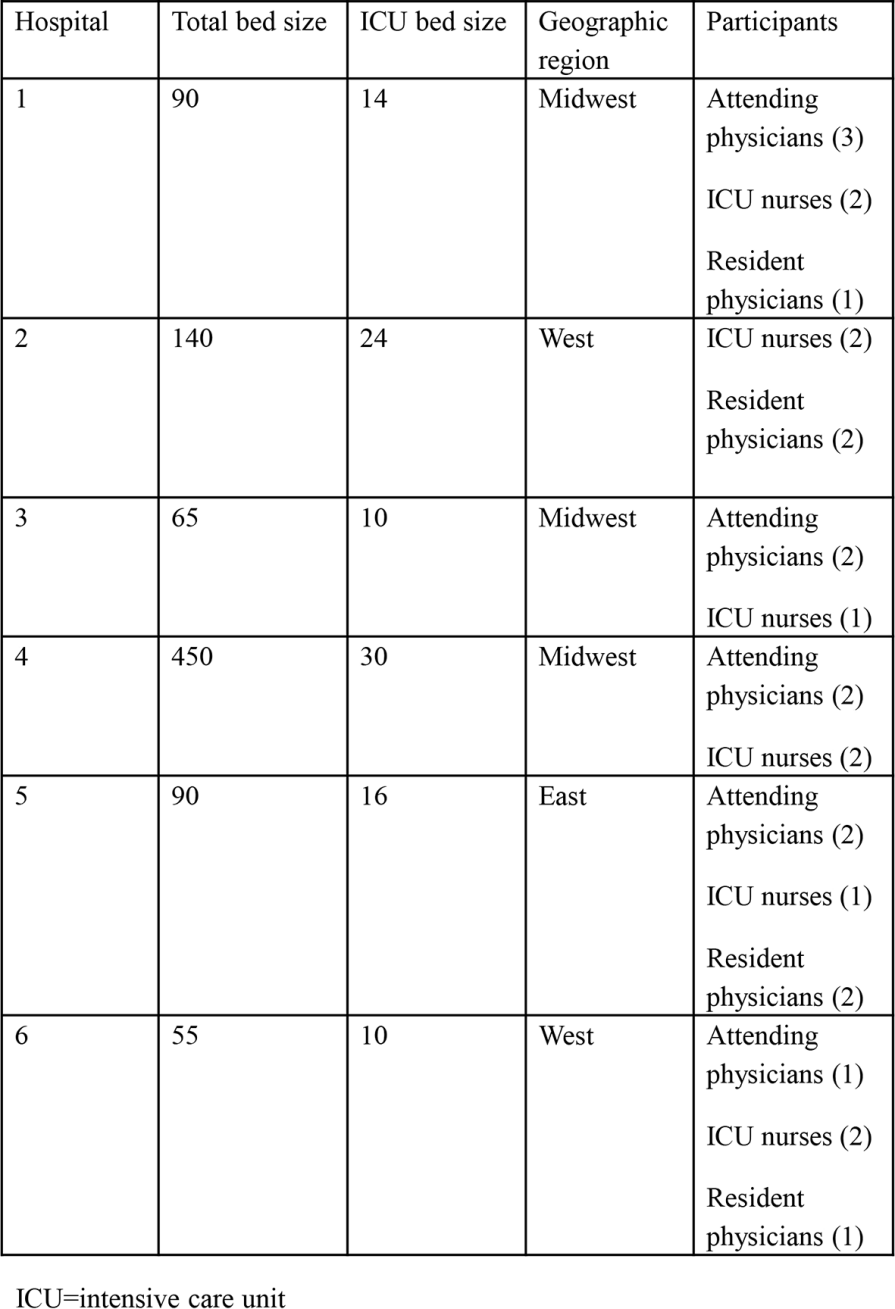
Site and participant (n=26) demographics

When asked how someone might know if a physician is competent or “signed off” in CVC insertion, four major themes emerged: (1) institutional policy; (2) competency by numbers; (3) documentation of competency; and (4) perceptions of competency measures. See Table 3 for representative quotes.

### Institutional policy

Across institutions, participants discussed both policies and guidelines (or lack thereof) regarding the documentation of competency. Although most participants recognized the *existence* of a policy or guideline on independent CVC insertion privileges at their institution, few could provide a specific *description* of the policy. As an example, one nurse commented, “Yeah, I don’t know the specific criteria that they use, but they just don’t let anybody place it. I think they have to be a certain year in their residency, so it’s not like they just let an R1 [post graduate year - 1 resident] come and do that” (#16-Nurse-Male).

### Competency by numbers

Most of the participants who reported knowledge of an institutional competency policy reported the policy as being count-based, meaning that a specific number of supervised CVC insertions was required in order to demonstrate “competence.” However, among participants who were aware of the existence of an institutional policy, many could not specify or were unsure of the required number of procedures that defined competency. Such a lack of certainty was seen among all types of participants, regardless of their role within the system. Some participants noted being unaware of the specific information about the number of required CVCs, but indicated they knew how to obtain it. “There’s a certain number that they would need to do, and then they get certified. But that I would refer you to Medical Education, because they would know.. There’s a number and they would say, ‘OK. Now, I’m certified.’ But I don’t recall the number, because I’m not the one certifying them. But it is part of the standard medical education” (#11-Attending physician-Female).

Among those participants who identified a specific number required in order to demonstrate competence in CVC insertion, variation as to the expressed number existed, even within the same institution. Answers across institutions ranged from three to ten CVC insertions. Within a single institution, the largest range of perceived required CVC insertions was three to eight. Some participants cited the ACGME or ABIM as sources, while others referred to their residency program requirements, or speculated; “It’s not particular to the VA, but according to our governing body for residency, once you’ve performed five with the guidance of someone else, you’re considered certified to be able to independently place a central line” (#25-Resident physician-Female).

### Documentation of competency

Participants at all VAMCs reported having formal, computerized programs in place when asked specifically about a system for the documentation of competency; “If you really wanted to know how many they’d done, you could look at their procedural log. They have a Medhub® online procedure log” (#9-Attending physician-Female). Most participants appeared to be familiar with access to the documentation systems. Nurses reported using the systems as well, but some expressed concern regarding their accuracy; “And it’s never up-to-date. I mean literally. It is never.. It’s never up-to-date..” (#13-Nurse-Female).

Although all sites reported having formal documentation systems in place, many still appeared to rely on informal documentation (e.g., resident self-report) or made assumptions about an individual’s competence; “It’s usually sort of based on the good will of the resident, I guess, to be honest if they’re certified or not. Usually if you’re a senior resident in the ICU we’ve already ensured that you’re certified because you’re going to be here overnight. So you would need to be able to do a central line..” (#25-Resident physician-Female).

### Perceptions of competency measurements

Numerous participants provided personal insight regarding their perceptions of the adequacy of the competency measures in place at their institution. Several participants mentioned using experience (numbers) *and* comfort (confidence) as a starting point when assessing trainees CVC insertion competence. One attending noted. “I’d ask them how many have they placed before, or how comfortable they feel with their skill set. And that would give me an idea of how clearly I need to direct them through the procedure..” (#03-Attending physician-Male).

Furthermore, providers often appeared unconvinced of the efficacy of any known institutional policies in accurately validating procedural competency. Attending physicians were more likely to discuss this issue than residents or nurses, with a number of physicians expressing concerns regarding the adequacy of a count-based system; “Competent meaning they are allowed to do it by themselves after they’ve been supervised doing 3 of them.. Yeah, but I don’t know if that’s competent.” (#12-Attending Physician-Female).

**Table 3.**
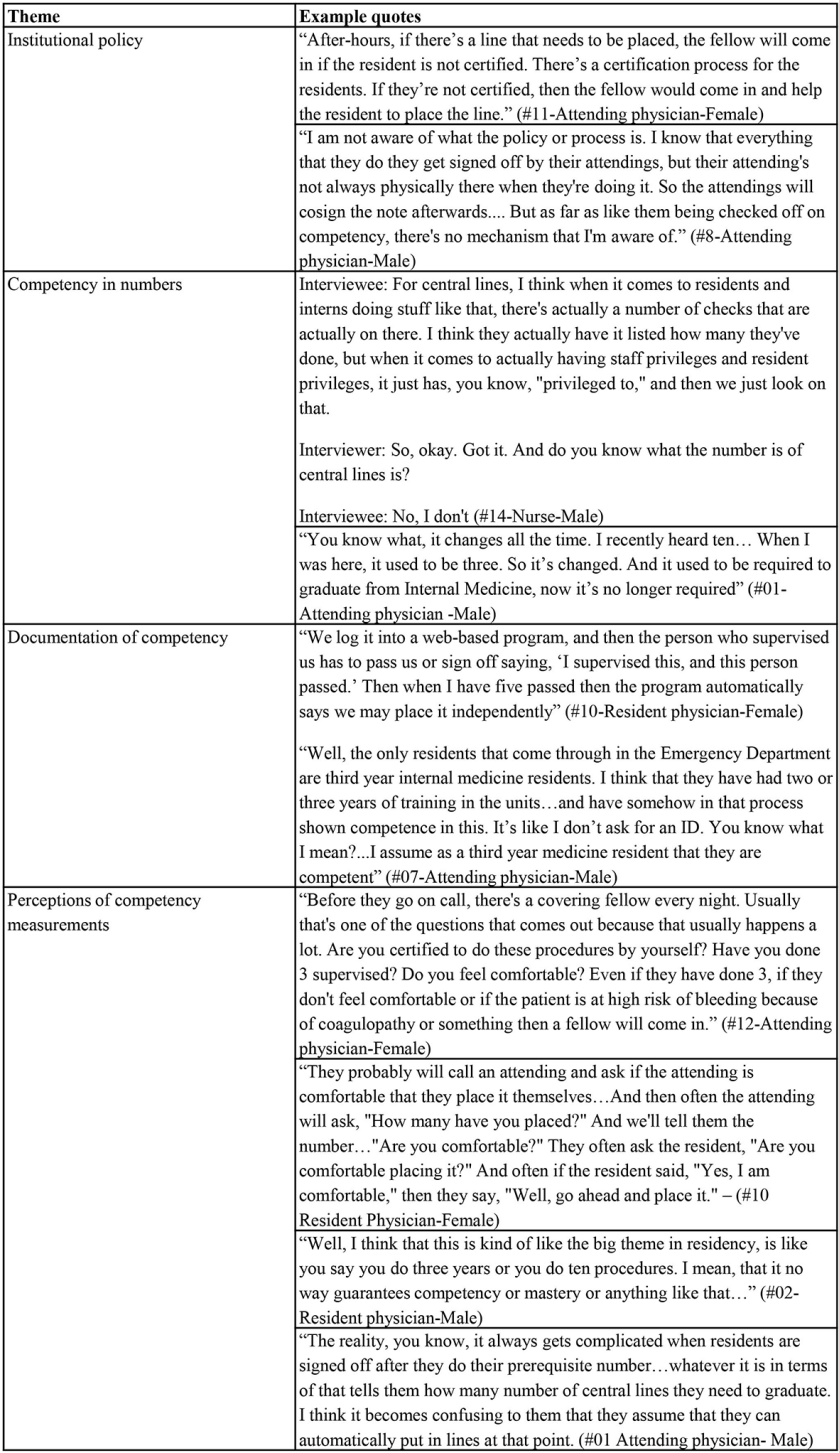
Themes and example quotes

## Discussion

Our study showed that participants had varying familiarity with institutional definitions of CVC insertion skill competence. A number of participants reported awareness of competency for CVC insertion being tied to requirement of a specific number of procedures, yet numbers identified both within and across institutions were inconsistent. Some participants reported a reliance on trainee’s confidence, in addition to the numbers, to assess CVC insertion competence. Finally, participants reported a concern about the reliability of these policies and existing measures of competence.

While the variation in responses we received may be due to institutional or training differences, it also may be due to the fact that within institutions the policies appear not to be well known among providers. An extensive review of 76 studies examining barriers to physician guideline adherence concluded that *lack of awareness* of guidelines negatively affected physician adherence ( Cabana et al., 1999), suggesting a first step to improving adherence be clear and ongoing communicaton of instiutional guidelines to ensure accurate knowledge of providers within each institution.

A relevant question is “which providers need to know the institutional competency requirements?” If a provider is unaware of a specific number needed to be identified as competent, but can identify which individual or group within an organization would know - is that sufficient? While it may not be necessary for all healthcare providers to be aware of hospitals’ CVC insertion competency guidelines, participants in this study were selected because they had been or would be training residents, are residents themselves, or would be assisting with CVC insertion at the bedside. These participants are on the front line of patient care and often need to make a split-second decision, perhaps in the middle of the night, as to whether or not the assembled team is truly competent to perform this potentially dangerous procedure. Identification, validation and dissemination of an accepted evidence-based standard of demonstration of competency, which can be agreed upon by accrediting organizations, hospitals and academic training programs, would provide a basis upon which to assess competency.

Within medical education, although we have seen significant progress, we are not so far beyond the era of “see one, do one, teach one.” Research has demonstrated trainee lack of confidence in their abilities/procedural skills; a survey of internal medicine residents revealed that residents needed more procedural experience than what was recommended by the ABIM in order to feel comfortable performing procedures on real patients such as CVC insertion, knee joint aspiration, lumbar puncture, and thoracentesis ( Hicks et al., 2000). Additional research examining the use of resident self-expressed comfort in performing bedside procedures demonstrated that more than 50% of resident physicians experienced discomfort in performing some aspect of bedside procedures ( Huang et al., 2006). Although the study noted that experience and supervision may serve to alleviate some discomfort, it also said that more formal and rigorous training in bedside procedures, including the use of simulation, could benefit trainees.

Furthermore, mere procedural experience does not ensure competence ( Wigton, 1996). Past research shows that residents, fellows, and attending physicians demonstrate uneven simulated skills performance in CVC insertion despite reporting a high number of CVC insertions in actual patient care ( Barsuk, Ahya, et al., 2009; Barsuk et al., 2016; Barsuk, McGaghie, Cohen, Balachandran, et al., 2009; Barsuk, McGaghie, Cohen, O’Leary, et al., 2009; McQuillan et al., 2015). A recent research synthesis noted weak positive associations between self-reported experience and simulated procedure performance; overall performance was poor even among the most experienced residents ( Barsuk, Cohen, Feinglass, McGaghie, & Wayne, 2017). Therefore, measures such as procedural volume are often unreliable and should not be used as the sole proxy for procedural skill. Moreover, reliance on procedural numbers as a surrogate of procedural competency is not evidence-based and may lead to improper patient care. Our interview participants may have recognized this danger as they specifically voiced concerns about the adequacy of determining competency only through a count-based system.

There may be some solutions to making the process of documenting competency easier and more consistent. First, institutional policies and required documentation must be clarified, while making documentation easy to use and access to ensure consistent expectations among all involved personnel prior to CVC insertion. Second, institutions need reliable and valid measures and interventions to improve performance ( Pronovost, 2010). Simulation is a solution that could provide trainees with the necessary tools to insert CVCs safely and competently ( Huang et al., 2006; Mema & Harris, 2016). Simulation-based education is recommended by the ABIM (“American Board of Internal Medicine Policies and Procedures for Certification, 2017”) and other subspecialty boards, and can be used in a mastery model where skills are measured against high achievement standards ( McGaghie, Siddall, Mazmanian, & Myers, 2009). SBML may be part of the answer to ensuring procedural competency determinations for medical regulatory bodies as well as hospitals and training programs. In SBML, trainees must meet or exceed a predetermined rigorous standard in a simulated environment that can be documented before performing the invasive procedure on patients ( McGaghie et al., 2009). Studies show that SBML is a more effective strategy than traditional clinical education alone ( Cook, Brydges, Zendejas, Hamstra, & Hatala, 2013; Didwania et al., 2011; McGaghie, Issenberg, Cohen, Barsuk, & Wayne, 2011a, 2011b), and SBML is an effective approach to ensure trainees and attending physicians consistently meet competency standards for the procedures they perform ( Barsuk, Ahya, et al., 2009; Barsuk et al., 2012; Barsuk, McGaghie, Cohen, Balachandran, et al., 2009; Barsuk, McGaghie, Cohen, O’Leary, et al., 2009; Wayne et al., 2008). The CVC SBML curriculum ( Barsuk et al., 2016) that was disseminated at the VHA as part of the larger dissemination study, has been shown to improve trainee CVC insertion skills, reduce mechanical complications such as arterial punctures, line malpositioning, and central line-associated bloodstream infections ( Barsuk, Cohen, et al., 2009; Barsuk et al., 2014; Barsuk, McGaghie, Cohen, Balachandran, et al., 2009; Barsuk, McGaghie, Cohen, O’Leary, et al., 2009). Moreover, use of CVC SBML resulted in a greater than seven-to-one return on investment ( Cohen et al., 2010). This approach to physician procedural competence aligns with the ACGME Milestones framework (“Accreditation Council for Graduate Medical Education Milestones”, 2016), and ensures policies and practices surrounding CVC insertion and other invasive procedures are uniform. By integrating SBML into the training curriculum, the policy regarding competency would be clear: all trainees must complete the SBML curriculum and reach mastery prior to being identified as “competent” to perform CVC insertion. We further recommend such a curriculum be made available, and preferably required, for any attending physician or other staff performing CVC insertion.

This study has several limitations. First, as our study was focused on the implementation of a training program within the VHA system, our results are limited by their focus on perceptions of individuals practicing within that system. However, many of our participants also were affiliated with local academic centers, and reflected on their perspectives within both systems. We recognize the limitation of generalizability of our sites, yet there is no reason to believe that the VHA system operates significantly differently from the private sector. Our sites were diverse both geographically and in size. Second, all study participants may have had vested interest in CVC insertion training, which may not be representative of practicing providers as a whole. However, it seems reasonable that individuals involved with training may be *more* likely to be cognizant of specific competency requirements, thus our findings of scant specific knowledge of competency requirements may represent an underestimate of general knowledge. Third, our findings may have been influenced by our own experiences and biases; we sought to minimize any such bias by having both a non-clinician and clinician as interviewers, and continuing interviewing until saturation was achieved. Finally, we went to VHA sites to perform SBML CVC insertion training, and recognize that we are suggesting SBML as a solution to many of the concerns identified by participants. However, other scholars have also identified SBML as a solution to improve procedural performance ( Gauger et al., 2010; Kessler, Auerbach, Pusic, Tunik, & Foltin, 2011; Scott et al., 2008; Stefanidis et al., 2007; Zendejas et al., 2011).

In conclusion, this study shows that healthcare providers’ knowledge and perceptions about institutional requirements for competency of CVC insertion vary widely. Some providers who were aware of the competency requirements also raised concerns about the reliability of current measures. The definition of competency must be expanded beyond the numbers to include rigorous training and assessments, where clinicians have the opportunity to acquire these critical skills in a consistent and reliable format, regardless of institution. Integration of SBML for CVC insertion offers a rigorous and reliable mechanism to train and assess procedural competency, and provides an accepted and safe process for residents and front-line providers to learn to perform procedures competently and provide safer patient care.

## Notes On Contributors

**Elaine R. Cohen, MEd** is a research associate, Department of Medicine, Northwestern University Feinberg School of Medicine, Chicago, Illinois

**Jeffrey H. Barsuk, MD, MS** is professor, Department of Medicine and Medical Education, Northwestern University Feinberg School of Medicine, Chicago, Illinois

**Joelle R. Hertz, MS** is a graduate student, Eastern Washington University, Cheney, Washington.

**Diane B. Wayne, MD** is vice dean, education and the Dr. John Sherman Appleman professor of medicine, Northwestern University Feinberg School of Medicine, Chicago, Illinois

**Yasuharu Okuda, MD** is associate professor, The Simulation Learning, Education and Research Network (SimLEARN), Veterans Health Administration, Orlando, FL, United States.

**Debi Mitra, MD** is instructor, Division of Hospital Medicine, Northwestern University Feinberg School of Medicine, Chicago, Illinois

**William C. McGaghie, PhD** is professor, Department of Medical Education, Northwestern University Feinberg School of Medicine, Chicago, Illinois

**Kenzie A. Cameron, PhD** is research professor, General Internal Medicine and Geriatrics, Medical Social Sciences and Preventive Medicine, Northwestern University Feinberg School of Medicine, Chicago, Illinois

## Declarations

Drs. Barsuk and Wayne received consulting fees from Medical Error Reduction and Certification, Inc. (MERCI). Drs. Barsuk, and Wayne and Ms. Cohen received royalties for the central venous catheter insertion simulation-based mastery learning curriculum from Northwestern University.
