# Supplementary material for: Healthcare Providers’ Awareness and Perceptions of Competency Requirements in Central Venous Catheter Insertion
Source: MedEdPublish (2016). 2018 Jan 15;7:12. [Version 1] doi: 10.15694/mep.2018.0000012.1 (PMC10699405; doi:10.15694/mep.2018.0000012.1)
Supplement: Peer review history

# Reviewer report: Reviewer response for version 1

**DOI:** 10.21956/mep.19490.r28791

**Published:** 2019-04-30

**Authors:** Ken Masters

**Affiliations:** 1 Sultan Qaboos University

This review has been migrated. The reviewer awarded 4 stars out of 5 An interesting paper dealing with healthcare providers’ awareness and perceptions of competency requirements in central venous catheter insertion. The authors have clearly identified the problem of the need to have competent providers in this area, compounded by a lack of consistency in guidelines and assessment processes and knowledge of these.The study has been well-conducted, with the sample size of 26 determined by data saturation, and then themes extracted from the verbatim interview material.Although the study is limited to one system only, there is enough of a variation in the settings in which the providers function, and this is enough to indicate that the results are at least partially generalisable beyond the narrow confines of the system. Overall, a useful (and somewhat worrying) paper that should encourage others to perform similar research in their settings, with a view to taking remedial action.

Reviewer Expertise:

NA

No decision status is available

# Reviewer report: Reviewer response for version 1

**DOI:** 10.21956/mep.19490.r28792

**Published:** 2018-01-18

**Authors:** Trevor Gibbs

**Affiliations:** 1 AMEE

This review has been migrated. The reviewer awarded 4 stars out of 5 An interesting paper that adds to our discussions on what really constitutes competency, how this is communicated in a structured and standardised manner and whether our model of competency applies within the real world. Although the paper relates to one specific competency, which I feel might belittle it somewhat in others' eyes, I think that its messages can be translated to other common competencies - perhaps the authors could have brought this out a little more in their discussion

Reviewer Expertise:

NA

No decision status is available
